# Low Concentrated Fractionalized Nanofibers as Suitable Fillers for Optimization of Structural–Functional Parameters of Dead Space Gel Implants after Rectal Extirpation

**DOI:** 10.3390/gels8030158

**Published:** 2022-03-04

**Authors:** Markéta Bocková, Aleksei Pashchenko, Simona Stuchlíková, Hana Kalábová, Radek Divín, Petr Novotný, Andrea Kestlerová, Karel Jelen, Petr Kubový, Peter Firment, Ján Fedačko, Taťána Jarošíková, Jiří Rulc, Jozef Rosina, Alois Nečas, Evžen Amler, Jiří Hoch

**Affiliations:** 1 Second Faculty of Medicine, Charles University, 15000 Prague, Czech Republic; stuchsim@cvut.cz (S.S.); andrea.kestlerova@lfmotol.cuni.cz (A.K.); evzen.amler@lfmotol.cuni.cz (E.A.); jiri.hoch@fnmotol.cz (J.H.); 2 University Centre for Energy Efficient Buildings, Czech Technical University in Prague, Trinecka 1024, 27343 Bustehrad, Czech Republic; petr.novotny.3@cvut.cz; 3 Department of Biomedical Sciences, University of Sassari, 07100 Sassari, Italy; 4 Faculty of Biomedical Engineering, Czech Technical University in Prague, 27201 Prague, Czech Republic; hana.kalabova@fbmi.cvut.cz (H.K.); jarostat@fbmi.cvut.cz (T.J.); rosina@fbmi.cvut.cz (J.R.); 5 Faculty of Physical Education and Sport, Charles University, 16252 Prague, Czech Republic; jelen@ftvs.cuni.cz (K.J.); kubovy@ftvs.cuni.cz (P.K.); 6 Department of Anaesthesiology and Intensive Medicine, FNsP J. A. Reimana Prešov, Jána Hollého 5898/14, 08181 Prešov, Slovakia; firment@fnsppresov.sk; 7 1st Department of Internal Medicine, Faculty of Medicine, Pavol Jozef Safarik University, Trieda SNP 1, 04190 Košice, Slovakia; jan.fedacko@upjs.sk; 8 Ambis College, Lindnerova 575/1, 18000 Prague, Czech Republic; jirka.rulc@centrum.cz; 9 Faculty of Veterinary Medicine, University of Veterinary and Sciences Brno, Palackeho tr. 1946/1, 61242 Brno, Czech Republic; necasa@vfu.cz; 10 Student Science, 19012 Prague, Czech Republic

**Keywords:** dead space, gel rigidification, fractionalized nanofibers, drug delivery system

## Abstract

Dead space after rectal resection in colorectal surgery is an area with a high risk of complications. In this study, our goal was to develop a novel 3D implant based on composite hydrogels enriched with fractionalized nanofibers. We employed, as a novel approach in abdominal surgery, the application of agarose gels functionalized with fractionalized nanofibers on pieces dozens of microns large with a well-preserved nano-substructure. This retained excellent cell accommodation and proliferation, while nanofiber structures in separated islets allowed cells a free migration throughout the gel. We found these low-concentrated fractionalized nanofibers to be a good tool for structural and biomechanical optimization of the 3D hydrogel implants. In addition, this nano-structuralized system can serve as a convenient drug delivery system for a controlled release of encapsulated bioactive substances from the nanofiber core. Thus, we present novel 3D nanofiber-based gels for controlled release, with a possibility to modify both their biomechanical properties and drug release intended for 3D lesions healing after a rectal extirpation, hysterectomy, or pelvic exenteration.

## 1. Introduction

Pelvic surgeries, especially those that are extensive such as extirpation of the rectum, hysterectomy, or pelvic exenteration, result in the development of dead space. This is often a reason for postoperative complications, such as bowel obstruction due to a relocation of small bowels into the dead space and their adhesion, hematoma, abscess, or perineal hernia. The fact that bony walls of the dead space are not compressible hinders the solution to most biomechanical problems of the filling materials. Optimal fillers should also stimulate cell proliferation, serve as antibacterial protection, and act against bowel (tissue) adhesion. Up to the present, autologous materials, predominantly omental and myofascial flaps, have mostly been used. The disadvantages of these matrices are that the omentum or muscle flaps’ length, number, and mobility are limited and often insufficient [1,2,3] and the associated surgical techniques are time-consuming and technically challenging [4,5,6,7].

Both synthetic and natural meshes have been tested to reconstruct the pelvic floor. Synthetic meshes like polytetrafluoroethylene or fully resorbable copolymer meshes are available in spray form only, and a risen thin layer is far from a suitable material capable of filling up the relatively large 3D dead spaces involved [8,9]. Moreover, since 2005, synthetic meshes have been practically replaced by biological meshes due to increasing infection concerns [10]. Biologic implants have been produced from porcine dermal collagen, intestinal submucosa, bovine pericardium, or human acellular dermal matrix. However, their application has not entirely solved the problems mentioned above [11,12,13,14]. These fillers suffer from a lack of prevention of tissue adhesion [15]. Newly developed materials, such as 4% icodextrin-alfa-1,4, have been found effective against adhesion; however, only the liquid forms have been found effective, with their effects disappearing within only four days.

The main aim of this study was to develop applicable fillers for the dead space meeting the following minimal requirements: 3D material with optimal biomechanical properties, minimal long-term adhesion formation, active against bacterial colonization, and promoting cell proliferation. To achieve these goals, agarose gels were chosen as an example of a biocompatible 3D scaffold, and fractionalized nanofibers were investigated as an active part of the composite. Agarose fillers are known in the field of aesthetic medicine [16], and several studies have proven their biocompatibility [17] and drug delivery applications [18], making agarose gels promising for surgical implants. However, agarose gels are usually too soft, and the optimal agarose concentration simultaneously showing an optimal rigidity and migration rate seems to be an obvious obstacle in abdominal surgery. Implant rigidity should be high enough to resist at least the pressure applied during laparoscopic surgery and viscous enough to retain in the space after surgery. The recommended pressure for laparoscopic operations varies between 8 and 16 mmHg [19,20]. These limitations could be solved by a suitable additive such as nanofibers. As the active component, nanofibers are also widely proven as a convenient biomaterial with promising applications in medicine [21]. A clear disadvantage of nanofibers is their fragility and 2D-like structure. These shortcomings are solved in our study. A combination of biocompatible hydrogels and fractionalized nanofibers functionalized with bioactive substances adhered on their surface or encapsulated in their core could trigger the development of a novel generation of dead space fillers.

## 2. Materials and Methods

### 2.1. Materials

Poly(vinyl alcohol) (PVA), Poly(ε-caprolactone) (PCL), Agar-Agar (powder BioScence–Grade, ROTH, Nuremberg, Germany), Standard agarose (powder, ROTH, Nuremberg, Germany), Fluorescein Isothiocyanate-Dextran (wt. 10,000, Sigma-Aldrich, St. Louis, MO, USA) (FSC), H3PO4, and Glyoxal (Sigma-Aldrich, MO, USA) were used. In vitro experiments were performed on immortalized BALB/3T3 mouse fibroblast cell lines obtained from the American Type Culture Collection (ATCC, Manassas, VA, USA). Mouse embryo fibroblast BALB/3T3 clone A31 cells (ATCC CCL-163) were cultured as adherent monolayers in plastic Petri dishes Nunc™ (Fisher Scientific, Waltham, MA, USA) in Dulbecco’s modified eagle medium (4 mM L-glutamine, 4500 mg/L glucose, 1 mM sodium pyruvate, and 1500 mg/L sodium bicarbonate, Sigma-Aldrich) supplemented with 10% (*v*/*v*) fetal bovine serum (Sigma-Aldrich, MO, USA) and antibiotic–antimycotic solution (Sigma-Aldrich, MO, USA). MilliQ water was used for the preparation of all aqueous solutions (Milli-Q^®^ IQ 7005 Water Purification System, Millipore, Billerica, MA, USA). All reagents and chemicals used in experiments were of analytical grade and were used as received.

### 2.2. Methods

#### 2.2.1. Preparation of Nanofibers

Nanofibers were prepared using the electrospinning method. PVA (5 and 40 KDa, 90% hydrolyzed, Merck) was dissolved in distilled water at 70 °C. A 10% *w*/*w* aqueous solution of PVA was used for electrospinning with the addition of phosphoric acid to lower pH and glyoxal as a PVA crosslinker. Electrospinning was performed by a spinner with a wire electrode (NanoSpider N500 Elmarco, Liberec, Czech Republic) and a wire collector covered with polypropylene nonwoven textile with an electrical field intensity of 25 kV/cm. The PVA nanofibers were crosslinked using glyoxal for 72 h at a temperature of 60 °C to prevent the dissolution of nanofibers in the wet environment.

PCL nanofibers were also prepared by electrospinning using the Nanospider Elmarco NS 500 spinner. PCL (Mn 45.000, Aldrich, MO, USA) was dissolved in chloroform at 23 °C. A 24% *w*/*w* solution was used for spinning. The fibers adhered to the nonwoven fabric throughout the spinning process.

Two types of nanofibers have been tested, namely PCL and PVA. PCL is based on a monomer caprolactone, a cyclic ester that is miscible, but hardly soluble in water, and possesses a seven-membered ring. This property determines its biological and medical application as a material with a long degradation time in aqueous environments, including the human body. PVA is a water-soluble synthetic polymer. It has the idealized formula [CH2CH(OH)]n. PVA is used in various medical applications because of its biocompatibility, low tendency for protein adhesion, and low toxicity. PVA is a hydrophilic water-soluble substance broadly used in medicine and cosmetics. It has a short degradation half-time in water, which is possible to regulate via degree of polymerization. 

#### 2.2.2. Fractionalization of Nanofibers

PVA and PCL fibers were cut into small pieces with scissors. They were then divided into two parts. 

Each working cycle included 10 grinding (1 min) and recooling periods (1 min). The applied impact frequency was 10 Hz. Nanofibers were ground in liquid nitrogen using a cryogenic grinder (Freezer/Mill 6870, SPEX SamplePrep, Metuchen, NJ, USA) with small vials.

#### 2.2.3. Visualization of Nanofibers and Diameter Determination

Nanofiber morphology of PCL and PVA nanofibers as well as fractionalized nanofibers were examined by electron microscopy. First, samples were sputtered with a thin gold layer using a rotary pumped coater (Q 150R S, Quorum, Laughton, East Sussex, UK). Magnification of the electron microscope (VEGA 3 SBU, Tescan, Brno, Czech Republic) varied from 500× to 2000×, and a secondary electron (SE) detector at an accelerating voltage of 10 kV was used. Subsequently, the average fiber diameter was measured for 50 randomly selected fibers using Tescan software.

#### 2.2.4. Gel Preparation and Its Functionalization

For preparing different sample mixtures of Agar-Agar, standard agarose was put into a heating nest (Heating Nest LTHS for 1000 mL), which was heated to 150 °C, and the gels were taken out whilst boiling. The gels were then poured into plastic petri dishes with diameters of about 3.5 cm, dept 0.5 cm, slowly cooled down to the laboratory temperature in a humid atmosphere, and stored at 5 °C.

Agar and agarose concentrations were selected based on the standard concentration for agar and agarose medium preparation. Other agarose concentrations (1% and 3%) were also prepared before the desired concentration (1.5%).

Agarose (0.15 g + PCL 0.15 g)/10 mL distilled water, (0.15 g + PCL 0.015 g)/10 mL distilled water and Agarose (0.15 g + PVA 0.15 g)/10 mL distilled water, (0.15 g + PVA 0.015 g)/10 mL distilled water–agarose gels were prepared using the above methods. However, 0.15 g of fibers per 10 mL of gel were added to the gel after cooling to approximately 60 °C. The reason for the reduced temperature was that nanofibers are not damaged at this temperature rate and their functionality is preserved.

#### 2.2.5. Determination of Mechanical Properties

The samples of gels were divided into 6 pieces with a sterile surgical scalpel measuring approximately 7 × 7 × 4 mm. Half of the pieces were used for the constant force load test-creep, and the other half for the destructive simple pressure test. The exact thickness of the individual samples was measured throughout the loading process. The unloaded cross section of each sample was measured using a calibrated photograph in the Autocad 2014 program. The instantaneous average cross section of the samples was calculated on the basis of the incompressibility condition, i.e., a constant sample volume, and Poisson’s constant was estimated as 0.5.

Quantities used:

E [Pa], modulus of elasticity from Kelvin model
η [-], viscosity from the Kelvin model
τ [s], relaxation time from Kelvin model
E_Y_ [Pa], Young’s modulus of elasticity of a simple pressure test
σ [Pa], internal tension
σ_S_ [Pa], limit of simple pressure test
ε [-], relative elongation of the simple pressure test
ε_S_ [-], maximum relative elongation of the simple pressure test
T [mm], thickness of unloaded sample
S [mm^2^], cross section of unloaded sample
V [mm^3^], volume of the unloaded sample

##### Destructive Simple Pressure Test—Estimation of the Elongation and Strength Limits and Young’s Modulus

**Figure 1:**
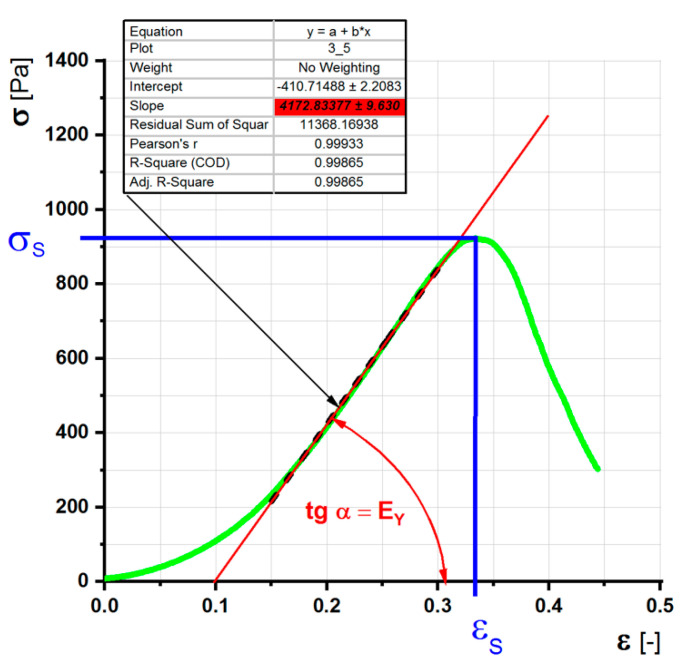
Determination of the limits of strength, elongation, and Young’s modulus of elasticity E_Y_ using the linear regression of the stress versus relative shortening curve.

The pressure test was determined using a Vertical Thermomechanical Analyzer TMA PT600 (Selb, Germany). The controlled variable was the deformation. All samples were compressed at a speed of 0.5 mm/s. The movement consistently started approximately 10 mm before reaching the sample and continued until the sample was compressed to a thickness of 1.1 mm, i.e., until it was completely crushed. The shredding machine was very oversized, so we considered the stress rate to be constant. This statement was also checked from the detection system record. The acquisition system stored data on time, sample thickness in the direction of stress, and tensile force throughout each test with a frequency of 200 Hz. From these records, the time course of the uniaxial stress σ [Pa] and the relative elongation ε [-] on time were determined. The stress was calculated from the applied force at each time point according to the instantaneous cross section of the sample. The cross section was calculated from the instantaneously measured sample thickness and assuming incompressibility, i.e., constant volume. From these calculated data, the functional dependences of the internal stress on the relative elongation σ (ε) were created. The mechanical characteristics of the individual gel samples were calculated from the graph of this dependence: Young’s modulus of elasticity E_Y_, yield strength σ_S_, and maximum relative elongation ε_S_. The complex modulus of elasticity E_Y_ was determined by linear regression (Figure 1).

##### Constant Load Test (Creep)-Estimation of Modulus of Elasticity and Viscosity

The tests were performed on a 1-axis loading device. The device was powered by a Kistler NCFH 10 actuator capable of developing a force of up to 10 kN with a working stroke of 200 mm. The drive was controlled by the Kistler MaXYmos system with a minimum step of 0.01 mm and precise control of the feed rate. Measurements of displacement, length, and elongation of the samples were provided by two independent incremental sensors with a step of 0.001 mm, which were in perfect agreement throughout the tests. We estimate the maximum absolute error of the sample elongation measurement to be ±0.01 mm. The tensile force on the sample was measured by two complementary connected 3-axis piezoelectric calibrated dynamometers Kistler 9317c. Their signal was processed by Dewetron DAQP ChargeB amplifiers (Grambach, Austria) and AD converter NI PCI 6281 (TX, USA). Position and force were recorded and analyzed in Dewesoft 7.1 software (Trbovlje, Slovenia); further postprocessing, calculations, analysis, and statistical processing were performed in MS Excel 2010 and OriginPro 2020. The accuracy of force gauges is given <1%, measuring range was ±8 N, and division size was <0.0001 N. Our estimation of force measurement error was ±2% of the magnitude of the quantity. The frequency of scanning all quantities was 2000 Hz; for analysis the data were reduced to a frequency of 200 Hz by selecting every 10 values. No hardware or software signal filters were used in the measurement or signal processing.

**Figure 2:**
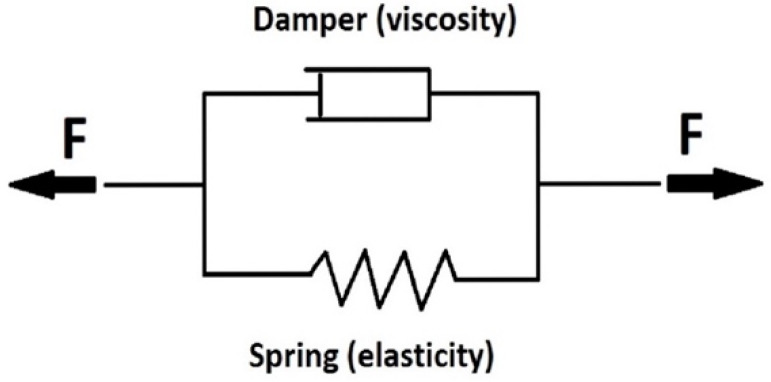
Scheme of Kelvin rheological model—parallel connection of spring and damper.

The samples were loaded with a constant force. The results were converted to a constant tension. To determine the rheological properties, the Kelvin viscoelastic model, a parallel connection of the damper and the spring, was chosen (Figure 2).

$$\sigma =E.\varepsilon +\eta \frac{d\varepsilon }{dt}$$

The measured relative elongation time curve was interpolated by the function:

$$\varepsilon =\frac{\sigma }{E}\left[1-exp\left(-\frac{E}{\eta }.t\right)\right]$$

#### 2.2.6. Degradation of PVA Nanofibers

PVA nanofibers were produced from a 10% PVA stock polyvinyl alcohol solution. For fluorescent labeling, a solution of PVA and 5-88 (Merck) at 1:1 *v*/*v* ratio was mixed with 0.042% Fluorescein Isothiocyanate-Dextran (FSC), (Sigma-Aldrich, MO, USA). Noncrosslinked nanofibers were prepared by electrospinning from a stock solution without any further treatment. Crosslinked nanofibers were prepared from the stock solution mixed with 0.6% H_3_PO_4_ and 0.4% Glyoxal (Sigma-Aldrich, MO, USA) and crosslinking was induced by 82 h incubation at 60 °C and 15% relative humidity. Nanofiber degradation was monitored by dissolving fluorescent labeled nanofiber scaffold in a 0.9% NaCl aqueous solution. Six identical fluorescent labeled samples with a weight of 0.280 g ± 0.003 g were put into glass bottles with 177 mL of 0.9% NaCl aqueous solution. The bottles with samples were placed in a shaking incubator with a shaking frequency of 60 Hz and a temperature of 37 °C. Aqueous solution samples of 100 µL from each glass bottle were taken at predetermined time intervals and analyzed using SYNERGY H1 (BioTeK, El Segundo, CA, USA) at an emission wavelength of 520 nm and excitation wavelength of 490 nm. Degradation of the functionalized PVA loaded with FSC was statistically determined using a calibration curve prepared by dissolving 0.007 g, 0.014 g, 0.028 g, 0.056 g, 0.084 g, 0.112 g, 0.14 g, 0.0168 g, 0.196 g, 0.224 g, 0.252 g, and 0.28 g of noncrosslinked fluorescent labeled samples loaded in 177 mL of 0.9% NaCl aqueous solution.

#### 2.2.7. Cell Proliferation Analysis

The MTS test was used to evaluate the influence of gels on cells proliferation. Samples comprising 2 mL of growth media (DMEM with FBS and ATB) were incubated for 48 h on the tested gels. 3T3 cells were seeded in 96-well culture plates at a density of 3000 cells/cm^2^. Each cell was supplemented with 20 µL of MTS substrate (Promega, Madison, WI, USA) and 100 µL of growth media. The cells were cultivated for 1 h under normal cell culture conditions (37 °C; 5% CO_2_; 95% humidity). Then, absorbance was spectrophotometrically measured (100 µL of product media at wavelength 490 nm) with a reference wavelength of 690 nm. Cells cultivated in a culture medium without incubation on gels were used as a negative control. Proliferation was measured on the 1st, 3rd, and 7th day of the experiment. Cell concentrations were determined by microscopy using a Burker chamber. On the 1st, 3rd, and 7th day of the experiment, samples were visualized using phase contrast microscopy (Optika, Ponteranica, Italy). Cells were seeded in a Petri dish with gel at a density of 3000 cells/cm^2^. The cells were cultivated under normal cell culture conditions (37 °C; 5% CO_2_; 95% humidity). After 72 h of cultivation, cells on gels were stained by the LIVE/DEAD^®^ Viability/Cytotoxicity Kit (L3224, Thermo Fisher, Selb, Germany) following protocol (Cell Viability Assays for Neural Stem Cells|Thermo Fisher Scientific, Selb, Germany).

## 3. Results

### 3.1. Gel Maturation Is a Key Step for Biomechanical Parameters

Agar and agarose gels were selected in this study as a basis for the preparation of 3D implants. All gels were prepared as described in the methods section. Importantly, maturation was found to be an essential step towards implant biomechanical property formation. We compared two freshly prepared samples of agar and agarose with maturated samples kept for one week at 5 °C under 100% humidity. The relative deformation ε at temperatures ranging between 31 °C and 45 °C was measured. A phase transition between 30 °C and 37 °C was identified in the agar sample (Table 1). This indicated that the material at the body temperature was too soft for an optimal implant. Agarose gel, however, showed much better characteristics, and a significantly broader phase transition makes it favorable for a clinical application. Clearly, such a gradual transition makes the implant more stable in case of temperature variation around 37 °C. In addition, we found that one-week-lasting maturation at 5 °C resulted in further broadening of the phase transition (Table 1), which makes the artificial implant even more biomimetic. Thus, we can apply maturation as a method for controlling biomechanical parameters.

### 3.2. Characterization of Fractionalized Nanofibers

**Figure 3:**
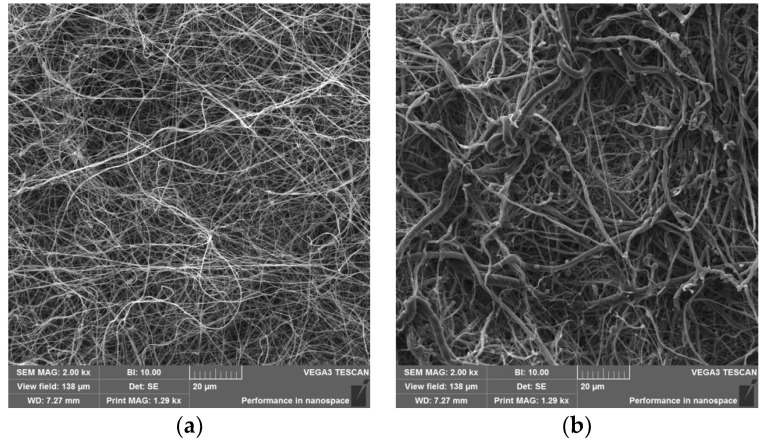

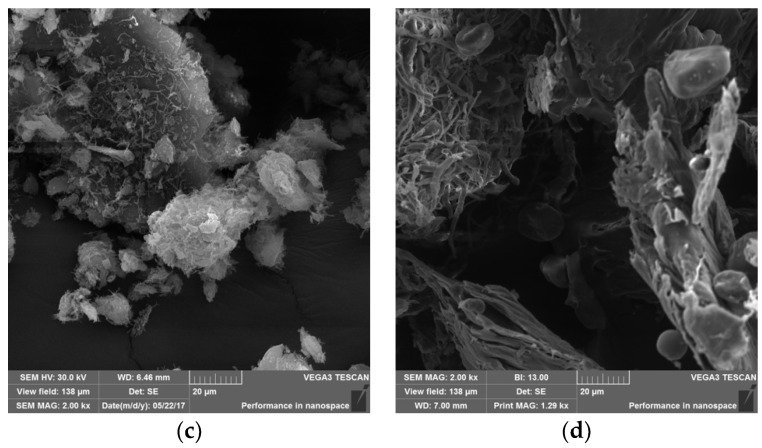
Scanning electron microscopy images of nanofibers. SEM of whole PVA (**a**) and PCL nanofibers (**b**); ground PVA (**c**) and ground PCL (**d**) nanofibers. Magnification 2000×.

**Figure 4:**
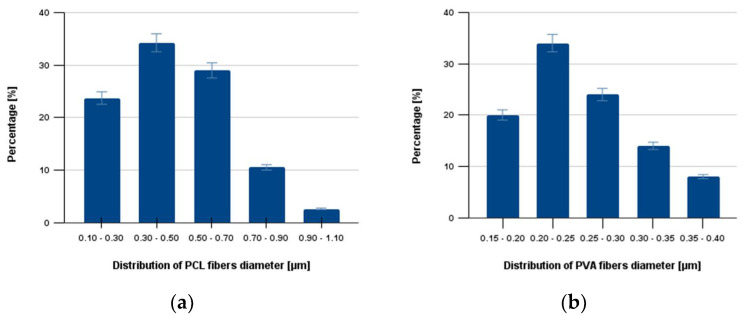
The average diameter of the PCL and PVA nanofibers. Subfigure (**a**) shows the average distribution of PCL nanofibers, and subfigure (**b**) describes the average distribution of PVS nanofibers.

Nanofibers from PVA and PCL were prepared by electrospinning as described in the methods section, and their structure was visualized by scanning electron microscopy (Figure 3a,b). SEM analysis of materials proved the fibrous morphology of both samples. Diameters of at least 50 different filaments were measured for each image and the resulting data were processed to yield the structural characteristics of the nanofiber mesh (Figure 4). 

Clearly, the diameter distribution showed significantly different structural characteristics for each nanofiber mesh. Different structural characteristics of nanofibers were observed for different polymers and for different physical parameters employed during the electrospinning process. Consequently, determination of structural characteristics belongs among the key parameters for reproducible production of the nanofiber-based drug delivery system. The average diameters of the PCL and PVA were determined immediately after electrospinning (Figure 4) and remained constant even after NaOH treatment. 

Nanofiber meshes prepared as described above are 2D-like materials due to their minimal thickness. This is hardly acceptable for filling large 3D lesions. To solve this problem, we prepared nanofibrous materials suitable for dispersion in liquids or gels. First, nanofiber mesh was fractionalized on micro-sized particles at a temperature below the gel phase transition. It was then mixed with a suitable gel. Such a 3D system maintained all the advantages of nanofibers, as fractionalization at liquid nitrogen temperatures yielded a fine powder with preserved nanofiber internal substructure (Figure 3c,d). 

The average diameter of the PCL and PVA nanofibers was determined from scanning electron microscopy images by TESCAN software. The average diameter of the PVA nanofibers was smaller than the average diameter of the PCL nanofibers because, with used fabrication technology, the higher viscosity of the used PCL solution limits the stretching of the jet during formation and therefore affects the diameter.

### 3.3. Fractionalized Nanofibers at Low Concentration Stiffen the Agarose Gels

**Figure 5:**
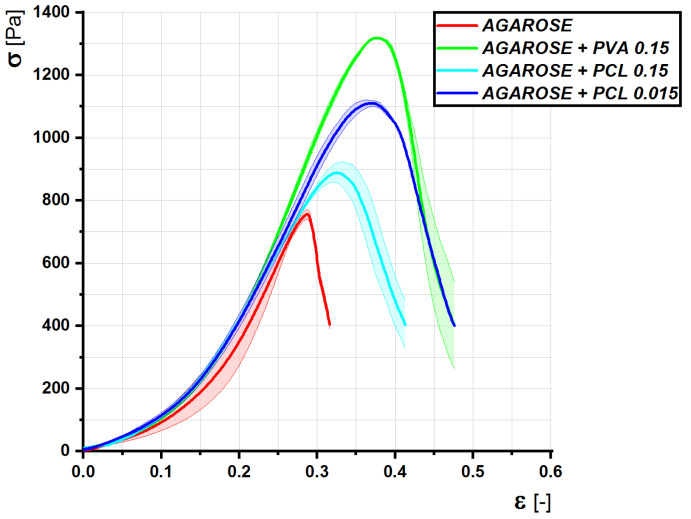
Dependence of the internal stress on the relative deformation under the loading of the gel by simple pressure. The thick line indicates the average of the measurements, the field standard deviation. The samples were compressed at a rate of 0.5 mm/s.

Gel functionalization was performed with fractionalized nanofibers to develop a novel generation of smart scaffolds with modifiable biomechanical and chemical properties. First, we tested the effect of fractionalized nanofibers on gel structural properties. The optimal description of biomechanical properties is a stress–strain diagram reflecting properties over a wide range of applied stress. We determined the stress–strain diagram for the agarose gel enriched with either PCL or PVA nanofibers (Figure 5). We proved that the addition of nanofibers (both PCL and PVA) significantly modified the stress–strain diagram, with PVA having a more profound effect. Tests were performed to determine the ultimate load capacity of the gels. The graph shows that the admixture of nanofibers significantly affects the yield strength and relative elongation. In the case of PVA, the yield strength increased by about 80% and allows the use of up to 10 mmHg of operating pressure, unlike agarose itself, which is destroyed at 6 mmHg. In the case of PCL, the increase is not as significant (50%), which corresponds to a destruction pressure of about 8 mmHg. In addition to an increase in the yield strength, nanofibers allow greater relative compression and elongation of the material from 0.29 to 0.37, an increase of about 25%.

**Figure 6:**
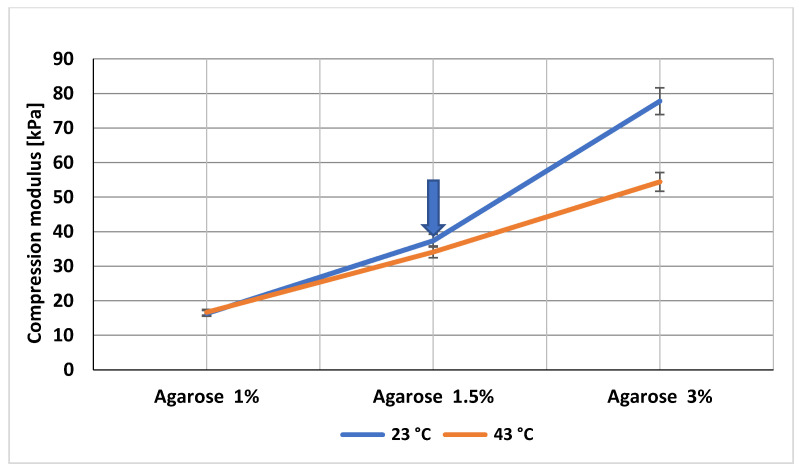
Dependence of the Young’s modulus of elasticity on agarose concentration.

Nevertheless, owing to the relatively narrow intervals of temperature and stress values to which the ideal implant should be exposed, the determination of the Young’s modulus of elasticity (i.e., at a single point of the stress–strain diagram) was chosen to be a sufficient experimental approximation. Gel deformation as a stress reaction was measured at 23 °C and 43 °C, and the Young’s modulus of elasticity was calculated. Figure 6 shows an almost linear dependence of Young’s modulus on agarose concentration at 23 °C. The same dependence was observed when the material was heated to 43 °C, leading to slightly reduced values of the Young’s modulus. 

Implant structure should resist at least the pressure applied during laparoscopic surgery. A set of gels withstanding the recommended pressure was prepared, and their biomimetic parameters were evaluated by surgeons. Based on the surgeons’ experience, the gel with Young’s modulus of elasticity characteristic for the 1.5% agarose was also identified as the most biomimetic gel resembling the native tissue macroscopically, as indicated by a blue arrow in Figure 6. Thus, we considered the pressure range affecting the optimal abdominal implant ranging from 10 mmHg to 14 mmHg, i.e., 1.3–1.9 kPa, and the gels with adequate rigidity were prepared and tested (Figure 6). 

Biomechanical gel properties belong indisputably among the key parameters of the optimal implant. The effect of fractionalized nanofibers on gel rigidity was considered as potentially significant and, thus, was carefully studied. 

The thick line indicates the average of the measurements, the field standard deviation. The samples were loaded with a constant force; the internal stress was calculated according to the instantaneous average cross section of the samples. The tests were performed in order to obtain the rheological properties of the gels, the modulus of elasticity, and the viscosity. These quantities were calculated using the Kelvin rheological model. From the graph and the calculated values, it can be deduced that the admixture of nanofibers causes a reduction in the modulus of elasticity and viscosity, more significantly in the case of PCL. The added gels will therefore be softer, more fluid, and at the same time withstand higher loads and deformations.

**Figure 7:**
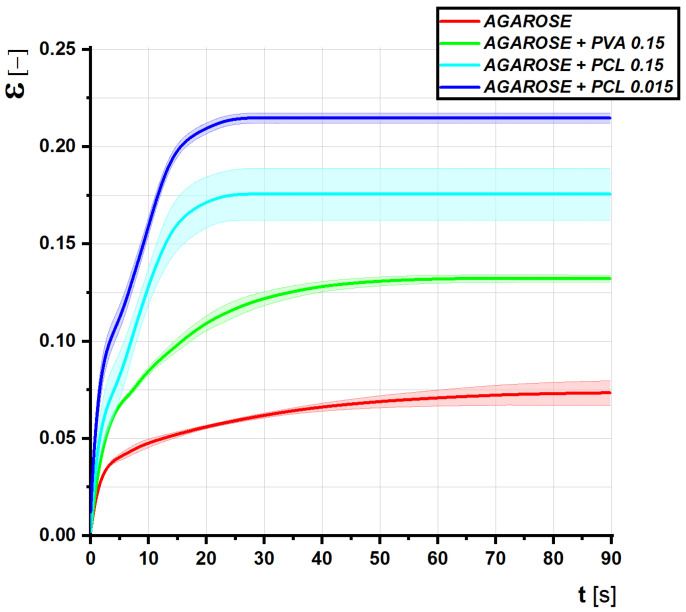
Graph of relative elongation versus time at constant internal stress for individual types of gel (creep).

Clearly, the presence of the fractionalized nanofibers significantly influenced the Young’s modulus of elasticity even at very low concentrations. The PCL nanofibers affected the relative elongation of gels more than the PVA (Figure 7). Both PVA and PCL fractionalized nanofibers, however, increased the Young’s modulus of elasticity at about 1.5% concentration. However, their effect was clearly observed already at a ten-times-lower scale, i.e., about 1.5‰ fractionalized gels.

### 3.4. Fractionalized Nanofibers Serve as a System for a Controlled Drug Delivery

First, fractionalized nanofibers were found as essential for gel rigidification. However, gel functionalization by degradable nanofibers could also open the door for the development of gels with the controlled release of encapsulated bioactive substances. Thus, the effect of nanofibers with a controlled degradation was examined and PVA nanofibers were used as a model system. Clearly, PVA as a hydrophilic substance is easily degradable in an aqueous environment. However, controlled crosslinking can prolong their degradation half-life in water. 

**Figure 8:**
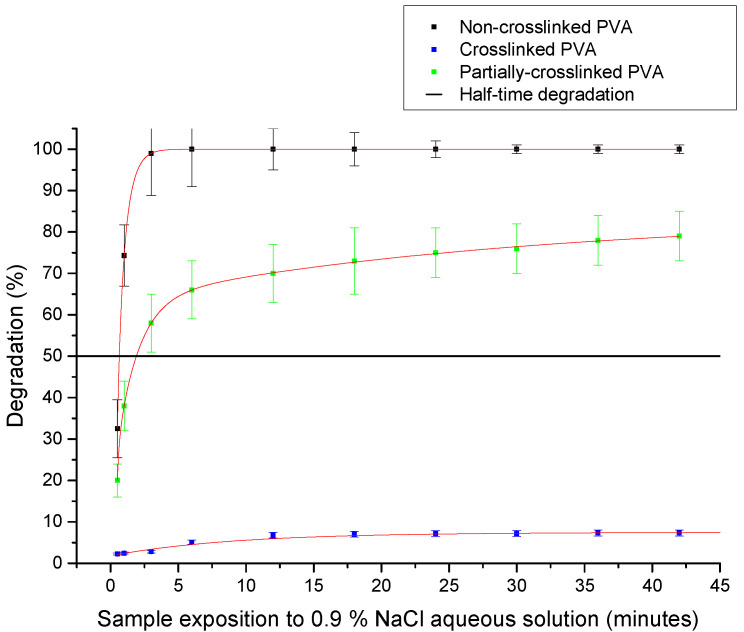
Degradation of crosslinked PVA nanofibers and noncrosslinked PVA nanofibers.

PVA nanofibers have been produced from polymers of different degrees of crosslinking, and their degradation was measured in distilled deionized water at a temperature of 36 °C on a vibrating heated platform with a vibrational frequency of 51 Hz. Fluorescein Isothiocyanate-Dextran was used as a marker for determination of the degradation half-time and nanofiber labeling was performed and analyzed as described in the methods section. Measured data clearly show that crosslinking can significantly prolong the degradation half-time (Figure 4). While the PVA nanofibers have been characterized with a degradation halftime shorter than 1 min, crosslinking prolonged this half-time by several orders. Noticeably, only 8% of the weight of samples with crosslinking modification were dissolved in 0.9% NaCl aqueous solution during 12 h. Thus, the system allows regulation of the degradation process on a very broad time window, as the half-time of degradation of our partially crosslinked sample clearly demonstrated (Figure 8). 

### 3.5. Biocompatibility Testing

Cell proliferation was used for testing of the fractionalized nanofibers’ biocompatibility. Both PCL and PVA fractionalized nanofibers were tested, and cells cultivated in a culture medium without incubation on gels were used as a negative control.

**Figure 9:**
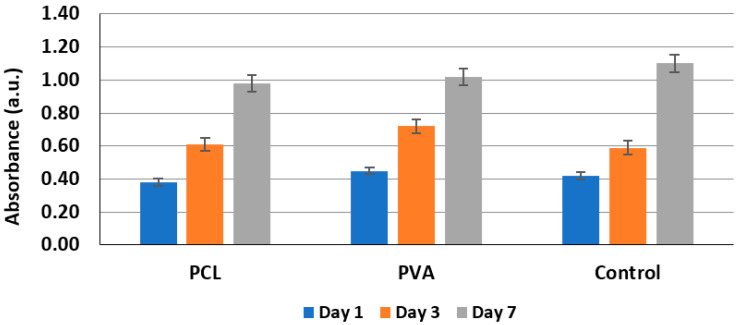
Cell proliferation analysis.

The cell cytotoxicity test did not show significant differences between the standard culture medium used for cell culture experiments, PCL-conditioned medium, and PVA-conditioned medium (Figure 9). Therefore, gels with PVA or PCL nanofibers were considered noncytotoxic and subsequently used for other cell culture testing. High cell viability was found on both gels’ material, more than 95% by the LIVE/DEAD^®^ Viability/Cytotoxicity Kit.

## 4. Discussion

### 4.1. Low Concentration of Fractionalized Nanofibers Can Significantly Modify Hydrogel Structural Parameters

Dead space after rectal extirpation in colorectal surgery is an area with a high risk of complications. Unfortunately, optimal methods for avoiding these complications have been missing. In the present study, we aimed to develop a novel 3D implant based on composite hydrogels with fractionalized nanofibers. Agarose and agar were chosen as the hydrogel standard for testing nanofiber effects. Despite the fact that the agarose and agar gels have been studied for a long time and have been shown as biocompatible, very little information about their exploitation in abdominal surgery has been reported [22,23]. Recently, research and the successful development of agarose fillers in aesthetic medicine and implantation surgery [24] has proven that agarose fillers cause minimal inflammatory response [16].

Medical application of nanofibers has several clear advantages. Among the more striking advantages is their biomimetic character, which resembles the structure of an extracellular matrix. In combination with a suitable gel, such a composite system has specifically required features, such as adherence to a target location, the possibility of microshaping, and the high surface area to volume ratio, which favors cell adhesion. Generally, prepared nanofiber meshes are usually very thin, 2D-like materials. This is hardly acceptable for filling large 3D lesions. To eliminate this problem, we prepared nanofibrous materials suitable for dispersion in liquids or gels. We used PVA and PCL, respectively, as polymers for nanofiber fabrication. Though PVA is approved by medical authorities, it is a slight irritant [25,26]. PCL is fully biocompatible [27]. We proved that gels could be used for minimizing the biocompatibility problems.

Nanofiber mesh (i.e., 2D-like material) was fractionalized to form micro-sized particles at a temperature below the gel phase transition. The microparticles were prepared with their average size comparable with the nanofiber mesh thickness. This step led to the production of an interesting material that could be mixed with the macro 3D systems (in our case, hydrogel on the agarose basis). Such a composite material has several advantages, their application to endoscopic or laparoscopic surgery undoubtedly being among the most important. The addition of nanofibers significantly affects the yield strength and relative elongation. In the case of PVA, the yield strength increased by about 80% and thus allowed the use of up to 10 mmHg of operating pressure [28,29,30]; unlike agarose itself, which is destroyed at 6 mmHg. In the case of PCL, the increase is not as significant; it is up to 50%, which corresponds to a destruction pressure of about 8 mmHg.

Nanofibers allow an increase in the yield strength and a greater relative compression of the material of about 0.39 in the case of PVA and about 0.35 in the case of PCL, i.e., an increase of 25 resp. 17 percent.

The results of determining rheological properties using creep are very interesting. With the admixture of nanofibers, the modulus of elasticity decreases significantly, and at the same time, the viscosity of the materials decreases significantly. Together with the simple pressure test results, these data prove that adding nanocomposites to the gels makes them become softer and more fluid, and at the same time withstand higher loads and deformations. In the case of the addition of PVA, the limits of possible stress increased more significantly, while the elasticity and viscosity increased only slightly. By adding PCL fibers, we did not achieve great durability, but the material softened more significantly.

### 4.2. Fractionalized Nanofibers Could Serve as a Superior Drug Delivery System

The unique properties of nanofibers, such as the high surface-to-volume ratio, porous structure, and nanoscale microarchitecture, make them ideal candidates for tissue engineering applications [31]. Surface modification of nanofibers is one of the simplest methods of their functionalization and could be used for adhesion optimization. In addition, nanofibers could be functionalized at their core. Several types of bioactive molecules have been successfully adsorbed into the surface of nanofibers, such as growth factors for the acceleration of tissue regeneration in hernia repair [32], antibiotics for the prevention of post-op adhesions [33], or liposomes loaded with growth factors to promote cell proliferation [34,35]. A clear advantage of nanofibers is their small diameter, facilitating the construction of microsystems able to accommodate cells, and the internal nanostructure serving as a cistern for the long-lasting release of bioactive molecules. These properties are unique and, combined with gels, could solve one of the main disadvantages of nanofibers in medicine: their pseudo2D structure, which is sufficient for acceleration of healing on surfaces, but insufficient for the healing of 3D lesions. 

In our study, fractionalized nanofibers were found to be an ideal additive for innovative 3D hydrogel-based implants. Besides their effect on biomechanical properties, low-concentrated nanofibers have been shown to be effective as a drug delivery system for a controlled release of encapsulated bioactive substances from their core. The scoring system was developed for the implant characterization and its further optimization, namely for a specific application (Appendix A).

In this study, we employed a combination of fractionalized nanofibers on pieces several microns or dozens of microns large. These pieces preserve their nano-substructure, which is important for cell accommodation, and their size simultaneously allows them obstacle-free migration throughout the gel. Thus, we could create three-dimensional nanofiber-based gels and exploit all the advantages of nanofibers for healing 3D lesions such as those following rectal extirpation. In summary, hydrogels enriched with fractionalized nanofibers functionalized bioactive substances (anti-inflammatory, growth stimulator, etc.) in their core.

## Figures and Tables

**Table 1:** Dependence-relative deformation on gel maturation.

Gel Composition Heated to 37 °C	ɛ =Δl/l (%)
Agarose 1.5% matured	14 ± 1
Agarose 1.5% fresh	18 ± 2
Agar-Agar 1.5%	24 ± 2

## Data Availability

The data presented in this study are available in the article.

## Appendix A

### Scoring for Gel Fillers

We found some gels to be suitable for filling up the space after the extirpation of the rectum. However, scoring is necessary to select the optimal gel from all gels tested and compare them with gels developed in the future. Selection of suitable gels for filling up the space after extirpation of the rectum is a difficult task from the medical and physical point of view, since each of them may be better in some respects and worse in others. The gel should meet the following minimal properties:

- biomimetic with optimal biomechanical parameters in the range between 36 °C and 41 °C
- high biocompatibility
- minimal invasion by prokaryotic cells
- optimal colonization by eukaryotic cells
- optimal surface parameters (optimal interface between the gel and tissue)
- optimal degradation halftime

Thus, based on the experience of the group leaders, the following scoring has been implemented:

- 0–5 points for optimal biomechanical parameters between 31 °C to 46 °C (most importantly 37 °C)
- 0–3 points for high biocompatibility
- 0–3 points for optimal colonization by eukaryotic cells
- 0–3 points for minimal invasion by prokaryotic cells
- 0–5 points for optimal degradation halftime
- Possibility of functionalization by bioactive substances
- Y/N extra points for optimal surface parameters (optimal interface between the gel and tissue) for some types of fillers
- Y/N extra points for including antibiotics

The maximum score is 20 points.
